# Machine Learning Models in Sepsis Outcome Prediction for ICU Patients: Integrating Routine Laboratory Tests—A Systematic Review

**DOI:** 10.3390/biomedicines12122892

**Published:** 2024-12-19

**Authors:** Florentina Mușat, Dan Nicolae Păduraru, Alexandra Bolocan, Cosmin Alexandru Palcău, Andreea-Maria Copăceanu, Daniel Ion, Viorel Jinga, Octavian Andronic

**Affiliations:** 1 Carol Davila University of Medicine and Pharmacy, Faculty of Medicine, General Surgery Department, University Emergency Hospital of Bucharest, 050098 Bucharest, Romania; florentina.musat@drd.umfcd.ro (F.M.); bolocan.alexa@gmail.com (A.B.); alexandru-cosmin.palcau@drd.umfcd.ro (C.A.P.); dr.daniel.ion@gmail.com (D.I.); octavian.andronic@umfcd.ro (O.A.); 2 Bucharest University of Economic Studies, Faculty of Cybernetics, Statistics and Informatics, 010374 Bucharest, Romania; andreea.copaceanu@csie.ase.ro; 3 Carol Davila University of Medicine and Pharmacy, Faculty of Medicine, Urology Department, “Prof. Dr. Th. Burghele” Clinical Hospital, 061344 Bucharest, Romania; viorel.jinga@umfcd.ro; 4 Innovation and eHealth Center, Carol Davila University of Medicine and Pharmacy Bucharest, 010451 Bucharest, Romania

**Keywords:** sepsis, prognosis, prognostic, artificial intelligence

## Abstract

**Background.** Sepsis presents significant diagnostic and prognostic challenges, and traditional scoring systems, such as SOFA and APACHE, show limitations in predictive accuracy. Machine learning (ML)-based predictive survival models can support risk assessment and treatment decision-making in the intensive care unit (ICU) by accounting for the numerous and complex factors that influence the outcome in the septic patient. **Methods.** A systematic literature review of studies published from 2014 to 2024 was conducted using the PubMed database. Eligible studies investigated the development of ML models incorporating commonly available laboratory and clinical data for predicting survival outcomes in adult ICU patients with sepsis. Study selection followed the PRISMA guidelines and relied on predefined inclusion criteria. All records were independently assessed by two reviewers, with conflicts resolved by a third senior reviewer. Data related to study design, methodology, results, and interpretation of the results were extracted in a predefined grid. **Results.** Overall, 19 studies were identified, encompassing primarily logistic regression, random forests, and neural networks. Most used datasets were US-based (MIMIC-III, MIMIC-IV, and eICU-CRD). The most common variables used in model development were age, albumin levels, lactate levels, and ventilator. ML models demonstrated superior performance metrics compared to conventional methods and traditional scoring systems. The best-performing model was a gradient boosting decision tree, with an area under curve of 0.992, an accuracy of 0.954, and a sensitivity of 0.917. However, several critical limitations should be carefully considered when interpreting the results, such as population selection bias (i.e., single center studies), small sample sizes, limited external validation, and model interpretability. **Conclusions.** Through real-time integration of routine laboratory and clinical data, ML-based tools can assist clinical decision-making and enhance the consistency and quality of sepsis management across various healthcare contexts, including ICUs with limited resources.

## 1. Introduction

Sepsis is associated with significant burden in modern intensive care units (ICUs), impacting patient outcomes, healthcare resources, and operational efficiency, with approximately 30% of ICU patients receiving this diagnosis [1,2]. Globally, sepsis affects 25 to 50 million people annually and is a leading cause of mortality [3,4]. The World Health Organization declared sepsis a global health priority in 2017, urging countries to monitor its incidence and outcomes closely [5]. Diagnosing and managing sepsis remains complex due to its heterogeneous presentation, the rapid progression of the disease, and the diverse ways patients respond to infection, which are driven by individual immune responses, comorbidities, and genetic factors [6,7,8].

Predictive survival tools can support clinicians by reducing uncertainty, allowing for more data-driven, informed decisions. These aim to take into account some of the numerous factors that could potentially influence the outcome in a patient with sepsis, such as age, underlying conditions, laboratory parameters, and acute physiological changes [9,10].

Identifying patients with a higher likelihood of survival enables clinicians to better stratify risk, target interventions, and allocate ICU resources effectively by prioritizing aggressive treatment for patients likely to benefit while also identifying those who might be better suited for palliative care [11].

Conventional scoring systems such as the sequential organ failure assessment score (SOFA), acute physiology and chronic health evaluation II (APACHE II, III, IV), simplified acute physiology score (SAPS II), sepsis severity score (SSS), acute physiology score (APS III), Oxford acute severity of illness score (OASIS), and logistic organ dysfunction system (LODS), while widely utilized in critical care, show marked limitations in predicting mortality and clinical outcomes among septic patients. Originally developed decades ago, these scoring models were calibrated to fit the patient populations and healthcare settings of their time [12,13]. However, both patient demographics and clinical environments have undergone significant changes, leading to a decrease in the accuracy and predictive validity of these models in contemporary sepsis care. Studies have highlighted that these traditional scoring systems struggle with both calibration and discrimination, resulting in poor accuracy when applied to current sepsis cases, particularly for in-hospital mortality prediction [13,14].

The SOFA score, for instance, remains one of the most commonly used tools for sepsis severity assessment due to its simplicity and ease of use. Yet, the SOFA score was developed based largely on expert consensus rather than robust empirical evidence [15,16]. Another critical limitation of these conventional scoring systems is their inability to account for the complex, nonlinear interactions between clinical variables that characterize sepsis [17]. Sepsis is a multifactorial condition involving complex interactions across inflammatory, immunological, and hemodynamic pathways, which can vary considerably between patients and even within the same patient over time. Conventional scoring systems are typically designed based on linear models, which restrict their capacity to model these dynamic, high-dimensional interactions. Consequently, they lack the adaptability to accommodate the heterogeneity and time-dependent nature of sepsis progression, often resulting in suboptimal predictions of clinical outcomes [18,19].

Despite extensive research into sepsis biomarkers, with over 250 identified to date, their utility in reliably predicting patient survival remains limited [20]. Prognostic biomarkers are utilized to predict the progression of sepsis, assess patient outcomes, and potentially guide therapeutic strategies. Traditional biomarkers with prognostic relevance are as follows [20,21]: lactate, D-dimers, cytokines (IL-6, IL-10), Pentraxin 3 (PTX-3), Adrenomedullin (ADM), Endothelial Cell-Specific Molecule 1 (ESM-1), S100 proteins, Plasminogen Activator Inhibitor-1 (PAI-1), N-terminal pro b-type Natriuretic Peptide (NT-proBNP), CD4+CD25+ Regulatory T Cells, noncoding-RNA (IncRNA CASC2, miRNAs), and sPD-Ll. Other emerging biomarkers such as Prokineticin 2 and Protein C show promising prognostic value but lack sufficient validation in diverse clinical settings. Additionally, while innovative prognostic markers like the CRP/albumin ratio (CAR) have been explored, their predictive accuracy in isolation remains insufficient for guiding clinical decision-making on patient survival.

Given the complex, multi-organ nature of sepsis and the vast amounts of patient data generated in intensive care units (ICUs), machine learning (ML) algorithms are well-suited for extracting relevant insights from extensive datasets and identifying subtle patterns that might not be apparent through traditional scoring systems or clinical judgment alone [22]. In sepsis care, where physiological variables change rapidly and clinicians face the challenge of synthesizing multiple layers of data, ML models provide an objective, consistent approach to predicting patient trajectories [23].

ML models can incorporate diverse data types; however, development of prediction tools based on available routine laboratory tests, demographic data, and basic vital signs would make them applicable across a broad range of healthcare settings, from high-resource ICUs to tertiary inpatient centers [24]. There is already ample evidence on the potential use of routinely collected ICU data for predicting complications, mortality, and improving prognostic models and patient classification via ML techniques [23]; however, the role of these data in ML-assisted predictions of sepsis outcome has not been systematically reviewed. According to a recent systematic review, demography, clinical history, and blood or urine parameters were employed in 79%, 73%, and 41% of the supervised ML-based diagnostic or prognostic studies [25]. In addition, previous research highlighted the frequent use of continuous measurements of vital signs in the ICU in the development of ML or natural language processing techniques for early diagnosis and prediction of sepsis occurrence [26].

Advanced machine learning (ML) techniques, including federated learning, explainable AI, and emerging innovations, are transforming sepsis care by addressing challenges in data integration, interpretability, and clinical application. Federated learning enables decentralized model training across institutions, preserving privacy while incorporating diverse datasets to create robust, globally applicable models. Explainable AI enhances the interpretability of complex ML models to clarify how variables influence outcomes. This transparency fosters trust and ensures accountability in high-stakes scenarios [27].

Complementing these, transfer learning and self-supervised learning reduce reliance on large, labeled datasets by leveraging pre-trained models or unlabeled data, enhancing predictions for specific populations or contexts. Advanced time-series models capture temporal patterns in patient data, improving risk prediction. Graph neural networks can analyze relationships between patients, treatments, and conditions, while reinforcement learning can simulate treatment strategies, thus personalizing interventions. Synthetic data generation addresses privacy and dataset imbalances, and multimodal learning integrates diverse data types, such as electronic health records and genomic data, for comprehensive insights. Techniques like causal inference may uncover sepsis progression pathways, aiding targeted interventions. Real-time federated edge computing supports localized predictions at the bedside, and dynamic risk models continuously adapt to evolving patient conditions [28,29].

The aim of this systematic review is to identify recent studies that explore the application of supervised and unsupervised ML models in prognosticating mortality outcomes for critically ill septic patients, using limited but routine electronic medical records data (i.e., vital signs, demographic data, clinical background, routine laboratory tests results), showcasing the potential of ML-assisted tools in settings with varying levels of ICU resources.

This research highlights the dynamic and rapidly evolving role of ML within critical care, recognizing both the opportunities it presents and the challenges that remain in fully harnessing this technology to optimize patient outcomes in the management of sepsis.

## 2. Materials and Methods

A systematic literature review (SLR) of publications in English from the past ten years was conducted. The search for references was executed in the PubMed database on 7 May 2024 using the algorithm outlined below:

(((sepsis[Title/Abstract]) AND ((prognosis[Title/Abstract])) OR (prognostic[Title/Abstract]))) AND (artificial intelligence [MeSH Terms]).

Studies were screened for eligibility using the Population, Intervention, Comparator, Outcomes, and Study design (PICOS) criteria (Table 1) [30]. The methodology for reviewing and selecting the studies followed the recommendations of the Preferred Reporting Items for Systematic Reviews and Meta-analyses (PRISMA) statement [31] and the Cochrane Collaboration, the Centre for Reviews and Dissemination [30]. Screening was conducted by two independent reviewers at both the title/abstract and full text levels, and a third independent reviewer resolved any disagreements. Data were extracted into a pre-determined, Microsoft Excel^®^-based template by one reviewer and checked by a second independent reviewer. Eligible studies reported the development of ML models for predicting mortality among adult patients admitted for sepsis, which incorporated commonly available laboratory measurements among the variables of interest. Elements extracted from each publication referred to study characteristics (study design, country, period, objective, population, and sample size), model characteristics (type, whether any comparisons were attempted, choice and proportion of the training and validation sets), variables used in the model, and results of the validation process (sensitivity, specificity, accuracy, F-measure, area under the receiver operating characteristic curve (ROC), C index, precision recall curve).

## 3. Results

### 3.1. Studies Characteristics

**Figure 1:**
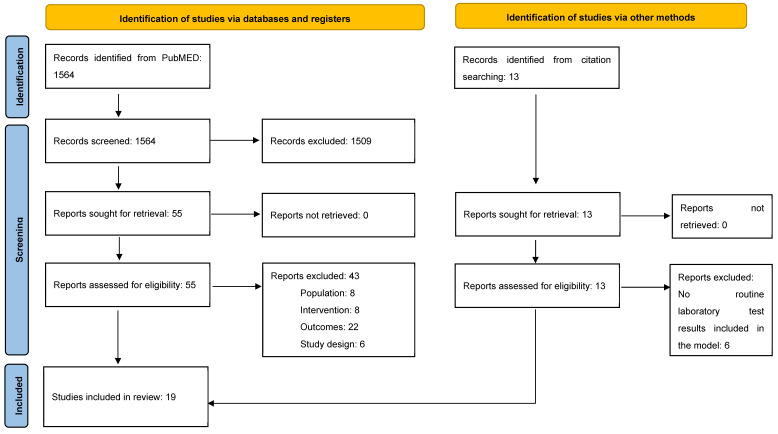
Literature review flow—PRISMA diagram.

A total of 1564 records were identified in PubMed, of which 55 were selected for review of the full text. In addition, six studies were identified through bibliographic cross-checking of previously published reviews. The detailed literature flow is described in Figure 1. Overall, 19 studies met the inclusion criteria and were included in the analysis. The studies included in this analysis employed various study designs, with the majority (16 studies) involving retrospectively collected data [8,9,11,12,15,22,32,33,34,35,36,37,38,39,40,41], while the remaining three were prospective observational studies [6,42,43].

The studies were conducted across various countries and utilized various databases (Table 2). The majority of studies (11 studies) [8,12,15,22,32,33,35,36,39,40,41] used United States (US)-specific data for the training and validation of models from the Medical Information Mart for Intensive Care III and IV (MIMIC-III and MIMIC-IV) and the Intensive Care Unit Collaborative Research Database (eICU-CRD). Two studies used datasets from China [8,34] and one study each had a population from Spain [42], Iran [9], the Netherlands [11], Colombia [6], and Poland [37], respectively. One study used data from both the US and China [8].

Both MIMIC-III and MIMIC-IV collected health related data from patients admitted to critical care units of the Beth Israel Deaconess Medical Center in the periods of 2001–2012 and 2008–2019, respectively. MIMIC-III [12,15,32,39] is the predecessor of MIMIC-IV [8,32,33,35,36,40,41]. Both are open access databases, and MIMIC-IV encompasses data from over 200,000 hospital admissions—providing, in addition to MIMIC-III, demographic information, vital signs, lab results, medications, detailed parameters for continuous renal replacement therapy, mechanical ventilation, and data from emergency and inpatient care before admission to the intensive care unit. However, data on post-discharge survival surveillance were not available. The Intensive Care Unit Collaborative Research Database (eICU-CRD) is a multicenter clinical database containing information on 795,780 patients admitted to 348 intensive care units across the US between 2014 and 2015 [8,22,35,41]. This dataset is available on PhysioNet (https://physionet.org/content/, accessed on 25 November 2024).

The objectives of these studies varied but commonly focused on mortality rates and prognostic factors in critical care settings (Table 2). Many studies aimed to identify mortality risks and prognostic indicators, with specific goals such as understanding 28-day survival rates, in-hospital mortality, and the impact of comorbid conditions such as diabetes or acute kidney injury (AKI). A notable subset of studies also compared traditional prediction models with newer ML techniques in terms of patient outcomes prediction accuracy.

The sample sizes of the studies ranged from 122 [37] to 21,680 participants [35], with a median sample size across all studies of 3937 participants, indicating a substantial variation in study scale and limited potential for generalizability of results in some studies.

### 3.2. Variables Used in the Models

On average, 45 variables (range 7 to 129) were considered per study, with high variability in the types and number of variables included in the development of each model. Several studies extracted data within the first 24 h of ICU admission [6,9,12,33,35,36,37,40,41,42]. This early data collection is critical, as it captures the initial severity of illness, which often dictates subsequent clinical outcomes. Observations in these studies varied, with some focusing on the worst values recorded [15,42] and others utilizing maximum, minimum, and mean values [35]. Interestingly, Guo et al. [32] and Taneja et al. [43] did not report specific timing for data extraction, highlighting potential variability in data collection protocols. Other studies [8,22] extended data collection to within the first 48 to 72 h, providing a broader temporal window for capturing critical clinical metrics. Additionally, van Doorn et al. [11] focused on a very early timeframe, collecting data within the first two hours post initial presentation in the emergency department, emphasizing the immediacy of data capture in acute care settings.

A descriptive analysis of the types of variables included in the models for predicting sepsis mortality was conducted. The variables were categorized into seven groups as follows: general information (i.e., demographic and clinical characteristics), vital signs, laboratory blood tests, arterial blood gas, comorbidities, treatment interventions, and others (Table 3).

**Figure 2:**
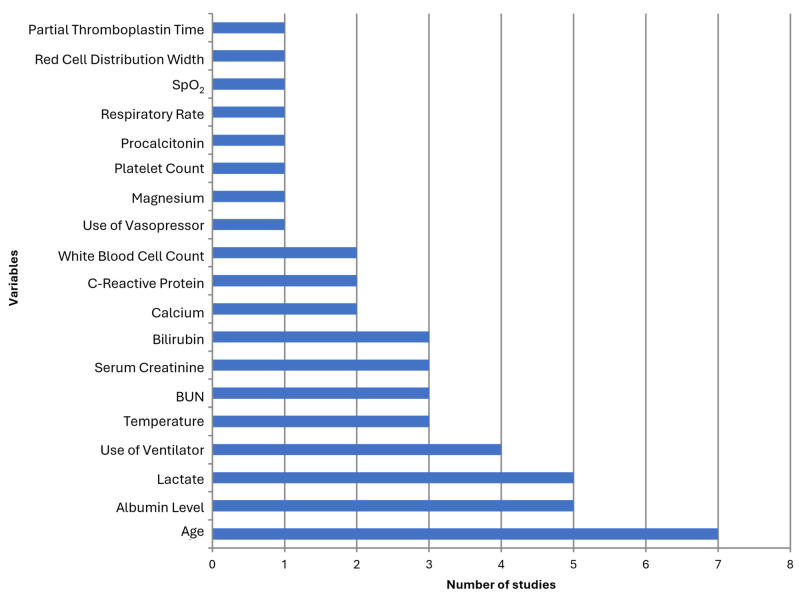
Prevalence of the most important variables for sepsis mortality prediction based on the extracted data. BUN—blood urea nitrogen. SpO_2_—blood oxygen saturation.

**Laboratory Blood Tests** were the most consistently included variables, featured in all 19 studies (Figure 2). These tests encompass a wide range of biochemical, hematological, and coagulation markers routinely used in clinical practice to monitor and assess patient health. Specific biomarkers such as C-Reactive Protein (CRP), Procalcitonin (PCT), Interleukin-6 (IL-6), D-dimers, and Fibronectin were included alongside routinely used markers in several studies, highlighting the role of these inflammatory and coagulation markers in sepsis prognosis (Table 3). In addition to utilizing a range of routinely measured clinical variables, Zhang et al. [41] uniquely incorporated several calculated ratios into their predictive model for sepsis mortality. These ratios include the Neutrophil-to-Lymphocyte Ratio (NLR), Lymphocyte-to-Monocyte Ratio (LMR), Platelet-to-Lymphocyte Ratio (PLR), Monocyte-to-HDL Ratio (MHR), and Neutrophil-to-HDL Ratio (NHR).

**Vital Signs** were included in 13 of the 19 studies, reflecting their significance in the early detection and monitoring of sepsis. Vital signs, including heart rate, systolic blood pressure, diastolic blood pressure, mean arterial pressure, temperature, and respiratory rate, are fundamental indicators of a patient’s immediate physiological state and are essential for identifying the onset and progression of sepsis.

**General Information** about the patient were utilized in 17 out of 19 studies. This category most frequently includes age and gender, and in some studies, weight, length, and ethnicity. The frequent inclusion of demographic data highlights the need to consider patient-specific factors when predicting sepsis mortality, as these can impact the disease trajectory and response to treatment.

**Arterial Blood Gas (ABG)** analyses were also included in 17 of the 19 studies. The most prevalent marker from the ABG panel included in the studies was lactate level. One study included only ABG measurements as prognostic variables: pH, Partial pressure of carbon dioxide (PaCO_2_), Partial pressure of oxygen (PaO_2_), Oxygen saturation (SaO_2_), Bicarbonate (HCO_3_), Base excess (BE), Lactate, and Glucose [22]. The high prevalence of ABG variables in predictive models indicates their importance in assessing the severity of sepsis and guiding therapeutic interventions.

**Comorbidities** were considered in 10 of the 19 studies, either as comorbidity scores (Charlson index, Elixhauser comorbidity index) or as specific conditions. Neurological diseases (including stroke, paraplegia, and cerebrovascular disease) were the most frequently included comorbidities (six studies), followed by diabetes (four studies). Cancer, renal disease (acute/chronic), chronic pulmonary disease, and metastatic cancer were included in three studies each. Comorbidities such as congestive heart failure, peripheral vascular disease, liver disease, obesity, and cardiac arrhythmia were present in two studies each. Less frequently included comorbidities were myocardial infarction, pancreatitis, recent surgery (within 2 weeks), use of immunosuppressants within the past 30 days, peptic ulcer disease, rheumatic disease, valvular heart disease, hypertension, hypothyroidism, coagulopathy, alcohol abuse, and depression (one study each).

**Treatment Measures**, such as interventions and therapies administered to patients, appeared in 12 studies. Mechanical ventilation and vasopressor administration were the most frequently included (four studies each). Antibiotherapy, blood transfusions, and renal replacement therapy were each included in two studies. Less frequently included measures were the use of anti-Xa agents and anti-thrombin.

The **SOFA, APACHE II, and SAPS II scores,** included in five studies, further underscore the importance of organ dysfunction severity in predicting sepsis mortality.

Additional variables less-often utilized were urine output, Glasgow Coma Scale, admission type (emergency, post-operative, ward), length of ICU stays, length of hospital stay, sepsis focus, germ class, and polymicrobial infection.

### 3.3. Types of ML Models and Their Performance

**Figure 3:**
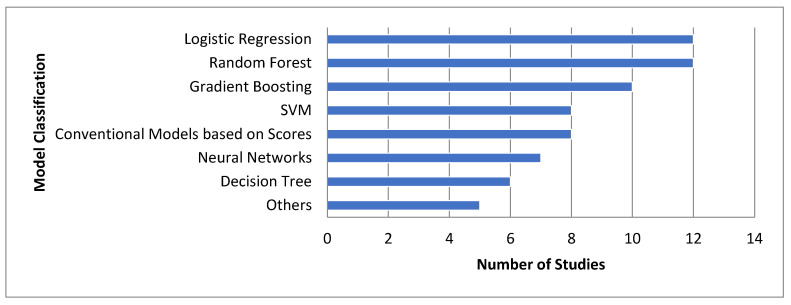
Model classification by count of studies. Others: DCQMFF—double coefficient quadratic multivariate fitting function, KNN—k-nearest neighbor, RFS—random survival forest, RVM—relevance vector machine, naïve Bayes.

The models used in the studies were categorized into seven distinct groups (Figure 3). The most frequently used were random forest (RF) models and logistic regression (LR), appearing in 12 and 11 studies, respectively, followed by gradient boosting models (10 studies) including variants such as XGBoost, gradient boosting machine (GBM), and LightGBM. Support vector machines (SVM) were developed in eight studies. Neural networks were used in seven studies, including specific deep learning algorithms such as convolutional neural networks (CNN) [32], multilayer perceptron (MLP) networks [9,11,35,40], long short-term memory networks (LSTM) [22], and artificial neural network (ANN) [6]. Decision tree models were employed in seven studies. Other models, such as double coefficient quadratic multivariate fitting function (DCQMFF) and relevance vector machine (RVM), were used in six studies. Conventional models based on scores, such as SOFA score and SAPS II, were used as comparators in eight studies. These statistics highlight the diversity of modeling approaches in the reviewed studies, with a notable preference for logistic regression methods and techniques like RF and SVM.

There were seven studies that investigated the use of a deep learning ML model, while all the other studies proposed traditional ML models. Guo et al. [32] developed a deep learning model based on a seven-layer convolutional neural network. The CNN model showed better accuracy, precision, recall, and AUC on the test cohort compared to traditional algorithms such as random forest, logistic regression, and LASSO regression. While traditional models have stricter requirements for the input data, the deep learning model is able to learn the relationship between the input variables and the corresponding class labels (outcome category) from complex nonlinear data. However, deep learning models have issues handling small datasets and, in these cases, their performance is not satisfactory. The CNN model proposed by Guo et al. showed an AUC of 0.909, compared to 0.807 obtained with the prediction SOFA score in the external validation cohort [32].

**Figure 4:**
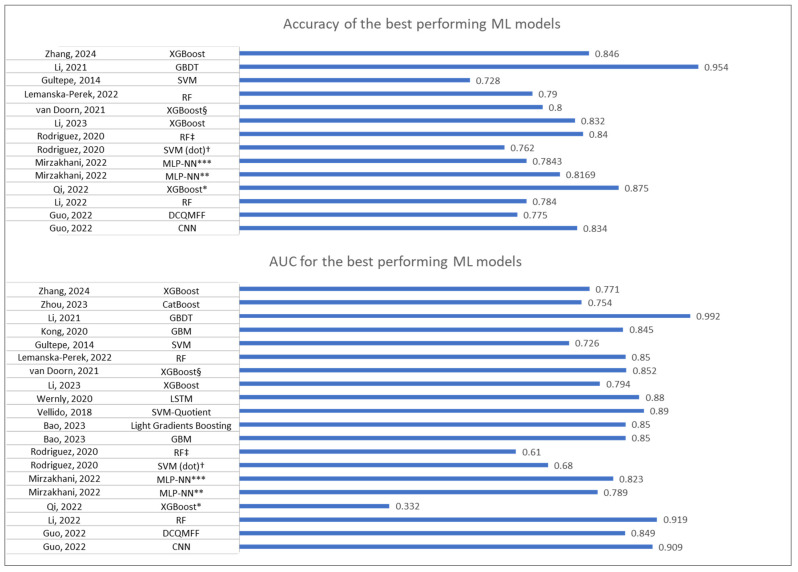
Accuracy and AUC at validation for the ML models with the best performance metrics from each study. * For the eICU-CRD dataset. ** Model developed with a subset of selected variables. *** Model developed with all available variables. ^†^ Model developed using the physiological and prognostic variables. ^‡^ Model developed using the clinical care variables. ^§^ Performance determined in comparison with predictions from physicians, abbMEDS, mREMS, and SOFA. CNN—convolutional neural network, DCQMFF—double coefficient quadratic multivariate fitting function, GBDT—gradient boosting decision tree, GBM—gradient boosting machine, LSTM—long short-term memory networks, MLP-NN—multilayer perceptron neural network, RF—random forest, SVM—support vector machine [6,8,9,11,12,22,32,34,35,36,37,38,39,40,41,42].

Accuracy and AUC at the validation step for the best-performing ML models selected from each study are presented in Figure 4.

#### 3.3.1. Accuracy

Accuracy is the proportion of true results (both true positives and true negatives) among the total number of cases examined. It is a primary measure of a model’s overall correctness [44]. In a medical context, a high accuracy in predicting sepsis-related mortality indicates that the model can reliably identify both patients who will survive and those who will die, which is critical for clinical decision-making and ensuring appropriate treatment [45]. The highest accuracy was reported by Li et al. (2021) [39], with a value of 0.954 using the gradient boosting decision tree (GBDT) model, suggesting the model’s effectiveness in predicting both survival and mortality, thereby reducing the risk of misdiagnosis and ensuring appropriate patient care. Conversely, the lowest accuracy was reported by Li et al. (2022) [34] with a value of 0.622 using the SVM model.

#### 3.3.2. Precision

Precision is the ratio of correctly predicted positive observations to the total predicted positives. It measures the model’s accuracy in identifying positive outcomes [46]. In a medical context, high precision ensures that most patients identified as high-risk indeed face a significant risk, thereby reducing unnecessary treatments and interventions. This is crucial in a medical setting where resources are limited and patient safety is paramount [47]. The highest precision was achieved by Qi et al. [8], with a value of 0.993 for a regression model, and by Li et al. (2021) [39], with a value of 0.948 for the GBDT model, suggesting that almost all identified high-risk patients are indeed at significant risk. On the other hand, Li et al. [36] (2023) reported the lowest precision at 0.425, suggesting low confidence in positive predictions and a higher likelihood of false positives.

#### 3.3.3. Recall

Recall measures the ratio of correctly predicted positive observations to all actual positives. It is crucial for identifying all true positive cases [46]. In a medical context, high recall is essential for ensuring that nearly all high-risk patients are identified, which is vital for timely and potentially life-saving interventions. Missing high-risk patients could lead to untreated conditions and increased mortality rates [47]. Qi et al. [8] achieved the highest recall of 0.989 with an RF model, indicating that the model correctly identifies 98.9% of patients who will die from sepsis, thus ensuring that nearly all high-risk patients are detected. In contrast, Li et al. [36] reported the lowest recall of 0.367, indicating that the model correctly identifies only 36.7% of actual mortality cases.

#### 3.3.4. F1 Score

The F1 Score is the harmonic mean of precision and recall, providing a balanced evaluation of these two metrics. It is particularly useful in the medical field to balance the trade-off between precision and recall [48]. In a medical context, a high F1 Score indicates that the model performs well in both precision and recall, ensuring a reliable prediction system that minimizes both false positives and false negatives [47]. Zhang et al. [41] reported the highest F1 Score of 0.96, demonstrating a well-balanced performance between precision and recall. Conversely, Mirzakhani et al. [9] reported the lowest F1 Score of 0.306, suggesting low balance with room for improvement in model performance.

#### 3.3.5. AUC (Area Under the ROC Curve)

AUC measures the model’s ability to distinguish between classes. A higher AUC indicates better performance and a greater ability to differentiate between patients who will survive and those who will die [48]. In a medical context, a high AUC is particularly important in a clinical setting as it indicates the model’s robustness in distinguishing between high-risk and low-risk patients, informing treatment decisions and improving patient outcomes [47]. Li et al. (2021) [39] reported the highest AUC of 0.992 for the GBDT model, indicating near-perfect discrimination. This high AUC suggests that the model is highly reliable for clinical application, where distinguishing between high-risk and low-risk patients is critical. In contrast, Qi et al. (2022) [8] reported the lowest AUC of 0.159 after external validation using a small dataset of patients from a different country (China) than that used for model training (US-MIMIC IV).

### 3.4. Handling Missing Data

Excluding cases or patients with missing data simplifies the dataset by removing incomplete entries, which can streamline the analysis process. However, this approach may lead to the loss of significant amounts of data, potentially reducing the statistical power and generalizability of the results. Additionally, there is a risk of introducing bias if the missing data are not randomly distributed, as the excluded cases might systematically differ from those included. Seven studies employed this approach [6,11,15,22,32,37,38].

When data are deleted based on a threshold for missingness, entries or variables with substantial amounts of missing data are removed, ensuring the retained data are more complete. While this helps maintain the quality of the dataset, it can also result in the loss of valuable information, particularly if the threshold is set too low. This approach can introduce bias if the missing data are systematic rather than random, affecting the representativeness of the dataset. Four studies applied this method [8,12,34,39].

Multiple imputation fills in missing values with plausible estimates based on the observed data, creating multiple complete datasets for more robust analysis. This method maintains the dataset size, preserving statistical power and reducing bias. However, it is computationally intensive and relies on assumptions about the missing data mechanism, which may not always hold true. If these assumptions are incorrect, the imputation may introduce inaccuracies. Five studies used multiple imputation [33,35,36,40,41].

Replacing missing values with the mean or median of the observed data is a simple and quick fix, preserving the dataset size and being easy to implement. However, this approach can distort the distribution of data, reduce variability, and potentially introduce bias, particularly if the missing data mechanism is not random. This method assumes that the mean or median is a reasonable substitute for the missing values, which might not be the case in all datasets. This approach was used in three studies [34,39,43].

Setting a threshold for missing data inclusion ensures that only variables with an acceptable level of completeness are included in the analysis. This method balances the need to retain as much data as possible with the desire to avoid the pitfalls of excessive imputation. However, setting the threshold too stringently may exclude important variables, impacting the comprehensiveness and richness of the analysis. This approach was noted in three studies [33,35,36].

Special methods, such as creating an additional variable to indicate the absence or presence of data, allow the model to account explicitly for missing data, potentially improving model performance. This approach retains all available data, avoiding the need for imputation or deletion. However, it adds complexity to the model and requires careful interpretation of the results, as the added variables might interact with other variables in unforeseen ways. This complexity necessitates a thorough understanding of the data and the relationships within them. An example of this approach is found in the study by van Doorn et al. [11].

### 3.5. External Validation, Reproducibility, and Interpretability

Among the nineteen studies, only five studies validated their proposed ML models on another cohort than that used for training and testing. Two studies chose as their external validation cohort an equally large database (eICU-CRD) as the one used for training and testing (MIMIC III or MIMIC IV) [8,32,35] (Table 4). The testing and external validation cohorts were from the same center in two studies: Guo, 2022 [32] used the MIMIC III dataset for training and testing and the MIMIC IV dataset for external validation; Mirzakhani, 2022 [9] used two groups of patients admitted to the same ICU of the Ghaemshahr’s Razi Teaching Care Center. The patient cohorts used for training datasets and external validation datasets were both from the same country in four of the studies [8,9,32,35]. Two studies [8,40] trained their proposed models on the US-based MIMIC-IV database and aimed to validate them using data from Chinese patients.

Interpretability of the ML models was addressed in 10 of the 19 studies by identifying the variables that are most important for the models’ decision-making. Only two studies provided open access to the code and/or the processed dataset. Although the studies that used MIMIC datasets cited them as publicly available resources, the authors did not share the subset that was ultimately used for the development of the models.

## 4. Discussion

The findings of this systematic review underscore the potential of ML models in enhancing sepsis outcome prediction compared to traditional scoring systems like SOFA and APACHE. These traditional models, widely used in clinical practice, often fall short in capturing the complex, nonlinear interactions between physiological variables critical to sepsis progression. ML approaches, particularly random forests, support vector machines, and neural networks, can outperform these conventional methods, providing higher accuracy in mortality predictions. Such ML techniques leverage extensive, high-dimensional datasets that include vital signs, laboratory values, and comorbidity indicators, enabling a more nuanced understanding of patient trajectories. This aligns with broader research indicating that ML’s adaptability to patient-specific data and real-time updates can significantly improve prognostic precision in critical care, though challenges remain in model interpretability, reproducibility, and the need for external validation.

### 4.1. Choice of Variables for Mortality Predictions ML Models in Sepsis

The choice of variables incorporated in the models is a crucial step ultimately influencing the performance. In Mirzakhani, 2022 [9], a sensitivity analysis was performed by incorporating in the MLP neural network model either all available variables or only selected variables that were significantly associated with the outcome of death in univariate analysis. This showed that for the model that used only the selected variables, state of consciousness, age, HCO_3_, bilirubin, creatinine, heart rate per minute, and hematocrit were the most important variables for predicting in-hospital mortality; meanwhile, for the model that used all available variables, blood urea nitrogen, temperature, platelets, respiration rate per minute, white blood count, creatinine, hematocrit, HCO_3_, and state of consciousness were the most important. The neural network model that incorporated only selected variables had better accuracy but lower AUC compared to the all-variables model, whereas the opposite could be observed for the CART decision tree model.

The core challenge with using biomarkers to predict survival lies in the inherent complexity and heterogeneity of sepsis. Sepsis pathophysiology is driven by a dynamic interplay of immune, coagulative, and metabolic responses, which vary significantly between patients. Biomarkers, in contrast, typically capture only isolated facets of this complex syndrome. As a result, they may reflect only a single aspect of the sepsis cascade, failing to provide a comprehensive picture of disease severity or progression. For example, elevated lactate levels may indicate tissue hypoxia but do not capture broader immunological or endothelial dysfunction, which are also critical to patient survival [20,21].

The most prevalent variables used for ML-based sepsis mortality prediction included age, lactate, albumin level, use of a ventilator, temperature, blood urea nitrogen, serum creatinine, and bilirubin. Other variables identified, though less frequent, included C-reactive protein (CRP), white blood cell count, calcium, use of vasopressors, procalcitonin, red cell distribution width (RDW), respiratory rate, SpO_2_, platelet count, magnesium, and partial thromboplastin time (PTT).

When comparing our identified variables with those included in the SOFA and APACHE II scores, several commonalities and differences emerged. Age, while not directly part of the SOFA score, is considered in the APACHE II score. Lactate, a strong indicator of sepsis and tissue hypoperfusion, is not included in either SOFA or APACHE II, yet its importance in sepsis prognosis is well recognized. The albumin level, indicative of poor prognosis in sepsis, is also absent from both scoring systems.

The use of a ventilator is indirectly assessed in the respiratory component of the SOFA score, while temperature is a component of the APACHE II score. BUN is indirectly included in the SOFA score as part of renal function assessment and directly in the APACHE II score. Serum creatinine and bilirubin are components of both SOFA and APACHE II scores. White blood cell count is included in the APACHE II score, whereas calcium, CRP, and procalcitonin are not part of either score despite their clinical significance. Variables such as the use of vasopressors and respiratory rate are crucial in both scoring systems, reflecting their importance in assessing cardiovascular and respiratory function. Other identified variables like RDW, SpO_2_, platelet count, magnesium, and PTT, while not included in the SOFA or APACHE II scores, provide additional valuable prognostic information. These findings suggest potential areas for enhancing existing clinical assessment tools by incorporating a wider range of prognostic indicators, thereby improving the accuracy and comprehensiveness of sepsis mortality predictions.

While all models incorporate routine laboratory tests, differences in the types and number of variables considered relevant are influenced by various factors. Study objectives, such as predicting in-hospital mortality or long-term outcomes, drive the inclusion of specific variables tailored to these aims. Data availability and quality also play a role, as certain datasets may lack detailed biomarkers or longitudinal metrics. Patient populations further contribute to variability. Studies focusing on subgroups such as elderly individuals or those with specific comorbidities require customized variables. Additionally, methodological choices, including feature selection techniques like LASSO or univariate analysis, and the type of ML algorithm (e.g., neural networks, random forests), can significantly affect which variables are prioritized. Differences in healthcare practices, technologies, and clinical priorities across regions and time periods further exacerbate this variability.

This heterogeneity has several consequences. It complicates cross-study comparisons and meta-analyses, limiting the ability to draw unified conclusions about the most important predictors of sepsis outcomes. It also impacts model generalizability, as models trained on context-specific variables may perform poorly when applied to new populations or settings. Overfitting is another risk, particularly when an excessive number of variables are included without robust validation, reducing model reliability. Furthermore, inconsistency in variable inclusion can lead to interpretability challenges and the potential introduction of bias.

To address these issues, future research should prioritize standardizing variable selection frameworks and emphasize external validation using diverse datasets. Reporting feature importance transparently can aid in identifying universally significant predictors, while multi-center collaborations can harmonize variable selection. These steps will enhance the robustness and applicability of ML models across different clinical settings and populations.

### 4.2. ML Techniques

The understanding of how the model works and the factors influencing the output are more challenging with increases in model complexity; however, these are necessary to show the model’s robustness, understand the parameters for use, and trust in the results it provides.

#### 4.2.1. Decision Trees

Decision tree-based models structure clinical and laboratory data into a hierarchical, rule-based format. They split datasets into branches based on influential features like age, comorbidities, and vital signs. Each branch leads to an outcome, with a leaf node often indicating a mortality probability. Decision trees prioritize influential clinical factors, such as low blood pressure or organ dysfunction, by positioning them near the root. This structure creates a series of “if-then” rules that guide clinicians through decision pathways, providing visual representations that highlight feature splits leading to different mortality probabilities.

Random forests, an ensemble method, enhance prediction accuracy by aggregating multiple decision trees. Each tree, trained on different patient data subsets, votes on mortality based on key features. The aggregated votes yield a final mortality probability score, ranking the importance of features across all trees for clinicians to visualize individual decision paths and rules.

The random survival forest (RSF) model, as described by Zhang et al. (2022) [33], ranks laboratory tests and comorbidities by their impact on 30-day mortality risk, aiding clinicians in prioritizing interventions for elderly patients. Similarly, the gradient boosting decision tree (GBDT) model used by Li et al. (2021) [39] and the CatBoost model (Zhou 2023) [40] offer risk scores and ranked feature importance for effective risk stratification and individualized predictions, guiding critical care decisions.

#### 4.2.2. Support Vector Machines (SVMs)

SVMs predict mortality by finding an optimal hyperplane that separates patients into different outcome groups based on clinical and laboratory variables. The algorithm identifies a hyperplane to classify patients into high-risk or low-risk groups, enhancing predictive performance through feature selection and kernel functions that transform data into higher-dimensional spaces. SVMs output mortality probabilities, helping clinicians tailor treatment strategies. Key data points, or support vectors, influence decision boundaries, with techniques like Recursive Feature Elimination ranking the importance of clinical features such as blood pressure, lactate levels, or comorbidities [49].

#### 4.2.3. Neural Networks

Neural networks learn complex patterns in patient data through multiple processing layers. They consist of an input layer with patient features, multiple hidden layers for data processing, and an output layer providing a mortality prediction. These models adjust weights through backpropagation, iteratively improving predictive performance [50].

Long short-term memory (LSTM) networks, a type of recurrent neural network (RNN), effectively analyze time-series data from arterial blood gases (ABGs). LSTMs capture temporal trends and dependencies, improving predictions of sepsis progression and mortality by considering vital sign dynamics. Despite their complexity and computational demands, LSTMs’ ability to handle sequential data often enhances accuracy [50].

#### 4.2.4. Logistic Regression

Logistic regression estimates mortality probabilities based on predictor variables such as age, lab results, and comorbidities. It outputs a probability score between 0 and 1, with each feature’s coefficient indicating its impact on the outcome. The model is trained on historical data to maximize the likelihood of correct predictions.

Common in medical research for binary outcomes like mortality, logistic regression provides interpretable results that quantify predictor impacts, often calculating odds ratios to indicate risk changes. This straightforward formulation aids in clinical decision-making. Logistic regression models vary by variable selection and construction techniques. Standard models use all available variables, while stepwise regression or LASSO refine predictor selection. Logistic regression is sometimes integrated into ensemble models for improved accuracy, though this can reduce interpretability compared to traditional models. Studies on specific populations, such as those with diabetes, highlight variations in predictor significance.

For instance, Qi (2022) [8] found that diabetic status significantly impacts predictive accuracy in sepsis outcomes, emphasizing the importance of managing blood glucose levels. Zhang (2017) [15] combined logistic regression with LASSO, thus reducing overfitting and enhancing prediction, identifying key predictors that outperformed traditional methods like SOFA and SAPS II scores.

In another study, Kong (2020) [12] integrated logistic regression into a broader ML approach to determine mortality predictors in a large ICU population, comparing baseline results with complex models like random forests and gradient boosting machines. This highlights logistic regression’s utility and adaptability while illustrating that ensemble methods may capture more complex variable interactions, offering enhanced predictive power.

### 4.3. Performance of ML Models

In clinical practice, a trade-off between sensitivity and specificity is often necessary. Enhancing sensitivity may lower specificity and vice versa. The optimal balance between these metrics depends on clinical goals and the consequences of diagnostic errors. For instance, in the case of sepsis, higher sensitivity is often preferred to avoid missing cases, even if this leads to some false alarms.

The study by Li et al. [39] stands out for its high performance across all metrics using the GBDT model. This model’s high accuracy, precision, recall, F1 Score, and AUC make it particularly suitable for clinical applications in predicting sepsis mortality, providing a robust tool for early intervention and treatment planning. High accuracy in Li et al. [39] indicates a model that can effectively predict patient outcomes, which is essential for reducing misdiagnosis and improving patient care. However, accuracy alone can be misleading in imbalanced datasets where the number of negative instances far exceeds positive ones. High precision, as seen in Qi et al. [8], is critical to ensure that most patients flagged as high-risk are indeed at significant risk, thereby avoiding unnecessary interventions. This is crucial in a clinical setting to optimize resource allocation and minimize patient burden. High recall, as demonstrated by Qi et al. [8], ensures that most at-risk patients are identified, which is vital for timely interventions. Missing high-risk patients could lead to untreated conditions and increased mortality rates. A high F1 Score, like that reported by Zhang et al. [41], indicates a well-balanced model that performs well in both precision and recall, ensuring a reliable prediction system that minimizes both false positives and false negatives. A high AUC, as reported by Li et al. [39], underscores the model’s robustness in distinguishing between patients at high risk of mortality and those who are not. This is particularly important in a clinical setting, where accurate risk stratification can inform treatment decisions and improve patient outcomes.

ML models show strong potential in predicting mortality for sepsis patients, often outperforming conventional scoring systems like APACHE II and SAPS II in sensitivity, while traditional models such as SOFA and SAPS II excel in specificity [9]. ML models can be more adaptable and precise by incorporating specific clinical factors, such as coagulation status, offering personalized predictions that static models may miss [32]. Studies also highlight that models like the LASSO score, which integrates routine clinical data, improve prognostic accuracy by capturing complex interactions among variables [15]. Unlike static methods, ML models benefit from continuous learning and updating, ensuring sustained accuracy as clinical practices evolve [6]. However, integrating these tools in healthcare requires careful ethical and practical considerations, viewing ML as an aid to, not a replacement for, clinical expertise [42].

### 4.4. Validity and Reliability of ML Models

Validation is an extremely important process that provides evidence as to whether the model can perform well on new data that it has never processed before. Internal validation refers to the process of splitting the overall dataset into a training dataset to be used for the model development and learning, and a test dataset to be used for measuring the performance of the model on data that it has not seen before. While internal validation is a widely used practice, it does not reflect the actual model robustness as the test data are highly similar to the training data and may overestimate the model performance on new data. As ML models are prone to overfitting, it is critical for models designed to be used in the context of assisting medical decisions to perform external validation by assessing the performance of the model using a different dataset than the training one. However, an external validation dataset obtained from the same hospital as the training may still be very similar to the training dataset. Performing external validation using datasets obtained in very different clinical settings than those present for the training dataset, such as from patients treated in another country than those involved in the model training, may be more challenging, but provides valuable insights as to the robustness and generalizability of the model.

Only a third of the studies identified in our review attempted external validation and only one used a dataset from a different country compared to the training dataset for external validation. Moreover, only one study provided evidence of the prospective use of the developed ML model in predicting the outcome for one patient with sepsis in the routine clinical context via a web-based application [37]. Similar to our findings, previous systematic reviews indicated that only a small proportion of ML models undergo external validation [25]. In addition, the majority of published AI-based research in sepsis diagnosis and prognosis is not followed up by examples of its use and results in daily clinical practice [25]. Online applications that prospectively collect prediction results and continuously improve database and model performance in real-time would be critical in determining the applicability and robustness of these tools.

### 4.5. Interpretability of ML Models

Model interpretability or explainability is a key feature of complex ML models, critical for their use in clinical practice. Half of the studies attempted to understand the factors influencing the models’ decisions by means of feature ranking methods. For neural network models, techniques like Layer-wise Relevance Propagation (LRP) or SHapley Additive exPlanations (SHAP) rank feature importance, offering insights into which features influenced risk scores. SHAP provides insights into ML model outputs using Shapley values from game theory to allocate “credit” to each variable based on its contribution to the prediction. Unlike direct effects in linear models, SHAP values capture the combined impact of each variable alongside others [51]. In the context of networks that categorize patients into risk groups based on mortality probability thresholds, positive SHAP values suggest a higher likelihood of death, while lower or negative values indicate a greater chance of survival. Substantial differences are observed between the sets of variables that have the highest influence on each model suggesting that other factors are involved in the model output. Internal mechanisms of deep learning algorithms are still not fully understood, and their results should be interpreted with caution and through the lens of the physician’s experience.

### 4.6. Evaluation of Reproducibility

The evaluation of reproducibility among the analyzed studies shows variability in adherence to best practices. Many studies leveraged publicly available datasets like MIMIC-III, MIMIC-IV, and eICU-CRD, enabling external validation and enhancing reproducibility. However, some relied on single-center or proprietary datasets, limiting accessibility and replication potential. Transparency in code sharing and methodological details was inconsistent, with only two studies explicitly providing their computational frameworks, which are critical for replication. External validation was a strong indicator of reproducibility, with studies testing models across multiple datasets demonstrating greater generalizability. In contrast, reliance on internal validation alone limited the robustness of findings. Performance metrics, such as AUC and sensitivity, were generally reported, but detailed documentation of preprocessing steps and experimental configurations was often incomplete. Studies incorporating explainable AI techniques contributed to transparency by elucidating the importance of individual features, thereby supporting reproducibility and interpretability. To improve reproducibility in ML research, future studies should prioritize open access to datasets and code, perform external validation on diverse datasets, and adhere to standardized reporting guidelines.

### 4.7. Comparison Between ML Models and Conventional Prediction Methods Based on Organ Dysfunction Scores

Conventional models that use scores such as SOFA, SAPS II, APACHE II, or APACHE IV for predicting outcome in patients with sepsis are constructed based on the assumption of a nonlinear relationship between patient characteristics and outcomes. ML models are designed to identify and leverage nonlinear correlations between input variables and output and have the potential to perform better than the traditional logistic regression algorithms. While logistic regression and APACHE II mortality prediction scores yield acceptable accuracy, their specificity is still limited [42].

Generally, ML models outperformed conventional logistic regression models, including those based on organ dysfunction scores [9,22,32,36,42]. Mirzakhani et al. [9] showed that the multilayer perceptron neural network models performed better in the external validation cohort in terms of accuracy and specificity compared to traditional scoring systems SOFA, SAPS II, APACHE II, and APACHE IV. Among the traditional scoring systems, APACHE II showed the best patient survival prediction, followed by APACHE II, while SOFA performed the worst.

Van Doorn et al. [11] compared the performance of the proposed XGBoost model with clinical judgment of acute internal medicine physicians and clinical risk scores mREMS, abbMEDS, and SOFA. Physicians were asked to predict 31-day mortality in a subset of patients not exposed to the ML algorithm based on clinical and laboratory data. While the sensitivity of the ML model was superior to that observed with the physicians’ predictions, abbMEDS, mREMS, and SOFA (0.92 vs. 0.72, 0.54, 0.62, and 0.77, respectively), its specificity was comparable (0.78 vs. 0.74, 0.72, 0.64, and 0.74, respectively).

### 4.8. Clinical Applicability: Enhancing, Not Replacing, Clinical Decision-Making

The ethical implications of using ML in healthcare are significant. Issues include data privacy, informed consent, and the potential biases in algorithms. For instance, there is a need to ensure that ML models do not perpetuate existing biases in healthcare systems [9]. The responsibility of healthcare providers must also be considered. ML tools should augment, not replace, clinical judgment. There is a need for continuous education and training for healthcare professionals to integrate ML responsibly, maintaining high ethical standards and ensuring that ML serves as a support tool rather than a replacement [9,52]. Mirzakhani et al. [9] compared ML models with traditional methods and found ML to be superior in prediction accuracy but emphasized the importance of clinician oversight. While these models can handle large datasets and identify patterns that might be missed by humans, difficulties in generalizing their use, ethical considerations, nuanced clinical understanding, and patient communication are some of the limitations that preclude relying solely on ML algorithms for decision-making.

## 5. Limitations

Several studies face the limitation of being conducted in a single center [6,9,12,15,33,34,41]. This design restricts the generalizability of their findings, necessitating validation across multiple centers to enhance external validity. The retrospective nature of studies introduces various biases such as selection bias and limits their ability to establish causality [9,11,22,34,35,36,38,41]. To address these issues, prospective multicenter studies are recommended.

Studies that are limited by their small sample sizes restrict the statistical power and generalizability of their findings [11,34,37,43]. Larger, more diverse samples are needed for more robust conclusions. Other studies highlight limitations in their ML models. These include the need for more comprehensive data, external validation, and challenges in interpreting “black box” models like neural networks. Future research should focus on improving model transparency and validation [9,11,15,33,34,35,40,41]. Some studies face issues with missing or incomplete data and the lack of dynamic data, limiting the accuracy and applicability of their models [35,39,41,43]. Incorporating more detailed and longitudinal data can improve model performance.

Another key limitation of the identified ML models is the reliance on relatively homogeneous training and validation datasets derived from the same country (often the US) and clinical setting. This may limit the global applicability and relevance of findings to other populations, particularly those in low- and middle-income countries where healthcare resources, patient demographics, and disease presentations often differ significantly. The issue lies in the potential for bias in model training, as algorithms are optimized on data that may not encompass the full range of variability seen in global healthcare settings. For instance, differences in disease prevalence, comorbid conditions, genetic predispositions, and socioeconomic factors across populations may influence both the manifestation of sepsis and the outcomes of treatment.

Furthermore, healthcare infrastructure and clinical practices, such as the availability of advanced diagnostic tools and therapeutic interventions, vary widely across regions. Models developed using data from resource-intensive settings may underperform in low-resource environments where such variables are absent or inconsistently recorded.

To mitigate this limitation, future research should prioritize the inclusion of datasets from diverse geographic regions and healthcare systems. Multi-center and international collaborations can help develop models that are more representative and robust. Additionally, stratifying analyses by region or population characteristics could improve understanding of how predictors differ across contexts. Expanding the diversity of training datasets will enhance the adaptability of ML models, ensuring they remain relevant and effective across a wider spectrum of patient populations and healthcare environments.

The abovementioned limitations are recognized by all the studies identified in our review. Rodriguez et al. [6] acknowledge that extreme values due to the biological variability in sepsis may skew the study’s results. Prospective and multicenter investigations are necessary to assess clinical applicability. Li et al. [36] point out that the reliance on the MIMIC IV database and retrospective design limits the ability to establish cause and effect, necessitating prospective randomized trials for validation. Taneja et al. [43] highlight that the study’s limitation to two clinical centers and its focus on a specific patient group (those with blood cultures ordered) reduce its generalizability. Including more geographically and socioeconomically diverse sites is crucial for broader applicability. Zhou et al. [40] acknowledge potential selection bias due to the use of a single database for the training set and stress the need for more external validations to confirm the study’s findings. Gultepe et al. [38] note that the mortality prediction model is limited by poor discriminability due to the lack of necessary temporal features in the time series data.

The strength of our review lies in the use of the systematic approach to comprehensively identify the evidence. However, several limitations of the review process should be noted. The literature searches were restricted to peer-reviewed journal articles published in English language, from the past 10 years, retrieved from only one database; thus, some potentially relevant studies may have been omitted. To mitigate the risk of missing relevant studies and validate our methodological approach, bibliographic lists of previously published systematic literature reviews were cross-checked for publications meeting our inclusion criteria.

## 6. Conclusions

ML holds promise for improving mortality prediction and outcomes in sepsis by providing more objective, accurate, and scalable insights than existing tools. Through real-time integration of routine laboratory and clinical data, ML-based tools can help bridge the gap between resource-intensive, high-tech ICUs and more standard care settings, ultimately enhancing the consistency and quality of sepsis management across various healthcare contexts.

ML-based tools should be integrated as supplements to clinical expertise, not replacements, ensuring that the human element in healthcare remains central to patient care. The future of ML in sepsis management lies in a collaborative approach where ML complements human expertise to improve patient outcomes, provided these technologies are developed and deployed with a balanced approach that respects both their potential benefits and ethical challenges.

## Figures and Tables

**Table 1:** PICOS criteria.

PICOS	Inclusion	Exclusion
Population	Adults with sepsis of bacterial origin, admitted to the intensive care unit	Patients with no sepsis Pregnant patients Sepsis with parasitic, viral, mycotic, or unknown origin Pediatric patients
Intervention/Comparator	Any intervention and comparator including no specified intervention	Not applicable
Outcomes and variables of interest	Characteristics and input variables of the ML model, including commonly used laboratory testing results among their inputs Performance parameters of the ML model such as accuracy, sensitivity, specificity, area under curve, etc. Prognostic factors for survival and mortality	Studies not reporting the input variables for the ML model Studies that did not include at least one commonly available laboratory measurement ML models not designed to predict survival/mortality outcomes
Study design	Supervised and unsupervised ML models designed to predict the survival/mortality outcomes and that included commonly used laboratory testing results among their inputs	ML models that did not include any routine laboratory testing results among their input variables

**Table 2:** Studies’ characteristics and objectives.

Reference	Study Design	Country and Databases	Period	Objective	Sample Size
Rodríguez 2021 [6]	Prospective observational, multicenter	Colombia	June 2014–February 2016	Prediction of in-hospital mortality in the ICU	2510
Guo, 2022 [32]	Retrospective multicenter	USA MIMIC-III; MIMIC-IV	2001–2012; 2008–2018	Prediction of 28-day survival rate Identify patients with high mortality risk Identify high-risk features associated with mortality	15,028
Zhang, 2022 [33]	Retrospective, single center	USA MIMIC IV	2008–2019	Prediction of 30-day mortality risk in elderly patients (65 and older)	6503
Li, 2022 [34]	Retrospective, single center	China	January 2013–January 2018	Prognostic factors related to death in patients with invasive candidal infection combined with a bacterial bloodstream infection	246
Qi, 2022 [8]	Retrospective, multicenter	USA; China MIMIC-IV; eICU-CRD; dtChina	NR	Prediction of in-hospital mortality in patients with diabetes	7001
Mirzakhani, 2022 [9]	Retrospective, multicenter	Iran	March 2017–September 2019	Comparison of conventional prediction models (SOFA, SAPS II, APACHE II, and APACHE IV) with modern ones (ANN and DT) in terms of the prediction of the survival	840
Bao, 2023 [35]	Retrospective, single center	USA MIMIC-IV; eICU-CRD	2008–2019; 2014–2015	Prediction of in-hospital mortality	21,680
Vellido, 2018 [42]	Prospective observational, single center	Spain	June 2007–December 2010	Identify prognostic factors for sepsis-related death Prediction of in-hospital mortality	354
Wernly, 2021 [22]	Retrospective, multicenter	USA eICU-CRD; MIMIC-III	2014–2015 2001–2012	Prediction of mortality within 96 h after admission Re-triage patients after 24–48 h of ICU treatment	13,634
Li, 2023 [36]	Retrospective, single center	USA MIMIC IV	2008–2019	Prediction of in-hospital mortality in adults with sepsis who developed AKI within 48 h of ICU admission	8129
Taneja, 2021 [43]	Prospective observational, multicenter	USA	February 2018–September 2019	Prediction of sepsis diagnosis Prognostic factors for hospital length of stay Prediction of 30-day mortality Prediction of 3-day inpatient readmission	350
van Doorn, 2021 [11]	Retrospective, single center	Netherlands	January 2015–December 2016	Prediction of 31-day mortality Comparison of ML models to internal medicine physicians and clinical risk scores	1344
Lemańska-Perek, 2022 [37]	Retrospective, single center	Poland	January 2018–December 2019	Prediction of 28-day mortality in patients treated for sepsis/septic shock	122
Gultepe, 2014 [38]	Retrospective, single center	USA	January 2010–December 2010	Determine the underlying relationships between lactate and patient outcomes, including mortality and sepsis Prediction of risk of mortality	151
Kong, 2020 [12]	Retrospective, single center	USA MIMIC-III	2001–2012	Prediction of in-hospital mortality	16,688
Li, 2021 [39]	Retrospective, single center	USA MIMIC-III	2001–2013	Prediction of in-hospital mortality	3937
Zhou, 2023 [40]	Retrospective, single center	USA MIMIC-IV	2008–2019	Prediction of in-hospital mortality in sepsis-associated acute kidney injury	16,154
Zhang, 2017 [15]	Retrospective, single center	USA MIMIC-III	2001–2013	Prediction of in-hospital mortality	3206
Zhang, 2024 [41]	Retrospective, multicenter	USA; Netherlands MIMICIV; eICU-CRD; The Amsterdam University Medical Centers	NR	Prediction of in-hospital mortality	3535

MIMIC—Medical Information Mart for Intensive Care; eICU-CRD—Intensive Care Unit Collaborative Research Database; SOFA—Sequential Organ Failure Assessment; SAPS—Simplified Acute Physiology Score; APACHE—Acute Physiology and Chronic Health Evaluation; ICU—intensive care unit; AKI—acute kidney injury; ANN—artificial neural network; DT—decision tree; NR—not reported.

**Table 3:** Categories of variables included in the models for predicting sepsis mortality and their frequency in the selected studies.

Variable	Frequency (Number of Studies)
Laboratory blood tests	19
Vital signs	13
General information *	18
ABG	18
Comorbidities	10
Treatment interventions	14
SOFA score	5
Ratios calculated **	1
CRP	3
Procalcitonin	3
Interleukin-6	1
D-dimers	1
Fibronectin	1

* General information refers to age and gender and, in some studies, weight, length, and ethnicity. ** Ratios calculated—Lymphocyte-to-Monocyte Ratio (LMR), Monocyte-to-HDL Ratio (MHR), Neutrophil-to-HDL Ratio (NHR), Neutrophil-to-Lymphocyte Ratio (NLR), Platelet-to-Lymphocyte Ratio (PLR). ABG—arterial blood gases. CRP—C-Reactive Protein.

**Table 4:** Machine learning models used for predicting sepsis and their performance.

Reference	Model	Accuracy	Precision	Sensitivity	AUC
Guo, 2022 [32]	CNN	0.834	0.825	0.818	0.909
	DCQMFF	0.775	0.764	0.754	0.849
	RF	-	-	-	0.533
	LR	-	-	-	0.605
	LASSO LR	-	-	-	0.567
	SOFA score	-	-	-	0.807
Zhang, 2022 [33]	RFS	C index 0.731	-	-	-
Li, 2022 [34]	LR	0.716	0.559	0.76	0.753
	RF	0.784	0.622	0.92	0.919
	SVM	0.622	0.465	0.8	0.777
Qi, 2022 [8]	LASSO LR	0.878 (eICU-CRD) 0.715 (dtChina)	0.993 (eICU-CRD) 0.790 (dtChina)	0.883 (eICU-CRD) 0.863(dtChina)	0.337 * (eICU-CRD) 0.201 * (dtChina)
	Bayes logistic regression	0.877 (eICU-CRD) 0.745 (dtChina)	0.983 (eICU-CRD) 0.818 (dtChina)	0.888 (eICU-CRD) 0.874 (dtChina)	0.290 * (eICU-CRD) 0.202 * (dtChina)
	Decision tree	0.865 (eICU-CRD) 0.763 (dtChina)	0.885 (eICU-CRD) 0.856 (dtChina)	0.972 (eICU-CRD) 0.867 (dtChina)	0.239 * (eICU-CRD) 0.159 * (dtChina)
	RF	0.886 (eICU-CRD) 0.760 (dtChina)	0.893 (eICU-CRD) 0.848 (dtChina)	0.989 (eICU-CRD) 0.874 (dtChina)	0.310 * (eICU-CRD) 0.162 * (dtChina)
	XGBoost	0.875 (eICU-CRD) 0.699 (dtChina)	0.875 (eICU-CRD) 0.699 (dtChina)	0.971 (eICU-CRD) 0.777 (dtChina)	0.332 * (eICU-CRD) 0.186 * (dtChina)
Mirzakhani, 2022 [9]	MLP-NN selected variables	0.8169	-	0.6667	0.789
	CART DT selected variables	0.5555	-	0.8333	0.3061
	MLP-NN all variables	0.7843	-	0.833	0.823
	CART DT all variables	0.7973	-	0.711	0.756
	SOFA score	0.6952	-	0.6667	0.76
	SAPS II	0.7095	-	0.6726	0.771
	APACHE II	0.733	-	0.739	0.803
	APACHE IV	0.711	-	0.736	0.785
Rodriguez, 2020 [6]	C4.5 decision tree clinical care variables	0.838	-	-	0.59
	RF clinical care variables	0.84	-	-	0.61
	SVM (ANOVA) clinical care variables	0.843	-	-	0.58
	SVM (dot) clinical care variables	0.845	-	-	0.58
	ANN clinical care variables	0.826	-	-	0.58
	C4.5 decision tree physiological and prognostic variables	0.639	-	-	0.53
	RF physiological and prognostic variables	0.741	-	-	0.65
	SVM (ANOVA) physiological and prognostic variables	0.708	-	-	0.69
	SVM (dot) physiological and prognostic variables	0.762	-	-	0.68
	ANN physiological and prognostic variables	0.706	-	-	0.69
Bao, 2023 [35]	SVM	-	-	-	0.75
	Decision Tree Classifier	-	-	-	0.75
	RF	-	-		-
	GBM	-	-	-	0.85
	MLP	-	-	-	-
	XGBoost	-	-	-	0.84
	Light Gradients Boosting	-	-	-	0.85
Vellido, 2018 [42]	LR-FA	-	-	0.65	0.78
	LR	-	-	0.64	0.75
	APACHE II	-	-	0.82	0.7
	RVM	-	-	0.67	0.86
	SVM-Quotient	-	-	0.7	0.89
	SVM-Fisher	-	-	0.68	0.76
	SVM-EXP	-	-	0.7	0.75
	SVM-INV	-	-	0.7	0.62
	SVM-CENT	-	-	0.7	0.75
	SVM-GAUSS	-	-	0.65	0.83
	SVM-LIN	-	-	0.62	0.62
	SVM-POLY	-	-	0.71	0.69
Wernly, 2021 [22]	LSTM (in eICU and MIMIC cohorts)	-	0.60 0.43	-	0.88 0.85
	LR (in eICU and MIMIC cohorts)	-	0.48 0.35	-	0.82 0.81
	SOFA score	-	0.23 0.24	-	0.72 0.76
Li, 2023 [36]	LR	0.822	0.572	0.608	0.73
	SVM	0.826	0.556	0.562	0.68
	KNN	0.793	0.429	0.367	0.601
	Decision tree	0.737	0.425	0.378	0.585
	RF	0.825	0.622	0.739	0.778
	XGBoost	0.832	0.66	0.793	0.794
	SOFA score	-	-	-	0.701
	SAPS II	-	-	-	0.706
Taneja, 2021 [43]	NR	-	-	-	
van Doorn, 2021 [11]	XGBoost	0.800 (in subset comparing with physicians, abbMEDS, mREMS, and SOFA)	0.387 (in subset comparing with physicians, abbMEDS, mREMS, and SOFA)	0.923 (in subset comparing with physicians, abbMEDS, mREMS, and SOFA)	0.852 (in subset comparing with physicians, abbMEDS, mREMS, and SOFA)
	LR	0.826	-	-	0.633
	RF	0.868	-	-	0.658
	MLP	0.842	-	-	0.723
	Acute internal medicine physicians	0.738	0.295	0.538	0.735
	abbMEDS	0.700	0.226	0.615	0.631
	mREMS	0.640	0.205	0.769	0.63
	SOFA score	0.740	0.303	0.721	0.752
Lemanska-Perek, 2022 [37]	RF	0.79	0.76	0.92	0.85
	LR	-	-	-	0.81
	GBM	-	-	-	0.78
Gultepe, 2014 [38]	SVM	0.728	-	0.949	0.726
Kong, 2020 [12]	LASSO LR	-	-	0.744	0.829
	RF	-	-	0.765	0.829
	GBM	-	-	0.771	0.845
	LR	-	-	0.76	0.833
Li, 2021 [39]	GBDT	0.954	0.948	0.917	0.992
	LR	0.821	0.723	0.776	0.876
	KNN	0.819	0.806	0.624	0.877
	RF	0.938	0.931	0.885	0.98
	SVM	0.86	0.828	0.749	0.898
Zhou, 2023 [40]	CatBoost	Internal validation: 0.75	Internal validation: 0.44	Internal validation: 0.75	Internal validation: 0.83, External validation: 0.754
	GBDT	Internal validation: 0.71	Internal validation: 0.40	Internal validation: 0.79	Internal validation: 0.82, External validation: 0.624
	LightGBM	Internal validation: 0.74	Internal validation: 0.43	Internal validation: 0.75	Internal validation: 0.82, External validation: 0.612
	AdaBoost	Internal validation: 0.79	Internal validation: 0.51	Internal validation: 0.65	Internal validation: 0.82, External validation: 0.595
	RF	Internal validation: 0.78	Internal validation: 0.48	Internal validation: 0.66	Internal validation: 0.82, External validation: 0.631
	XGBoost	Internal validation: 0.77	Internal validation: 0.46	Internal validation: 0.68	Internal validation: 0.81, External validation: 0.574
	KNN	Internal validation: 0.72	Internal validation: 0.41	Internal validation: 0.73	Internal validation: 0.80, External validation: 0.631
	MLP	Internal validation: 0.73	Internal validation: 0.41	Internal validation: 0.70	Internal validation: 0.79, External validation: 0.632
	LR	Internal validation: 0.73	Internal validation: 0.41	Internal validation: 0.71	Internal validation: 0.79, External validation: 0.709
	Naïve Bayes	Internal validation: 0.68	Internal validation: 0.37	Internal validation: 0.74	Internal validation: 0.76, External validation: 0.602
	SVM	Internal validation: 0.74	Internal validation: 0.43	Internal validation: 0.69	Internal validation: 0.76, External validation: 0.679
	SOFA score	-	-	-	Internal validation: 0.715
Zhang, 2017 [15]	LASSO score	-	-	-	0.772
	SAPS II	-	-	-	0.741
	APS III	-	-	-	0.737
	LODS	-	-	-	0.707
	SOFA score	-	-	-	0.687
Zhang, 2024 [41]	XGBoost	0.846	0.872	0.95	0.771
	SOFA score	0.844	0.854	0.91	0.702
	LR	0.836	0.838	0.884	0.703
	RF	0.832	0.845	0.853	0.677
	KNN	0.786	0.793	0.818	0.617
	Naïve Bayes	0.828	0.834	0.867	0.69
	SVM	0.83	0.825	0.858	0.658
	Decision tree	0.79	0.83	0.867	0.6

NN—neural networks; ANN—artificial neural network; CNN—convolutional neural network; GBM—gradient boosting machine; XGBoost—extreme gradient boosting; LightGBM—light gradient boosting machine; LASSO—least absolute shrinkage and selection operator; LSTM—long short-term memory; KNN—k-nearest neighbor; MLP—multilayer perceptron; LR—logistic regression; SVM—support vector machine models; GBDT—gradient boosting decision tree; RFS—random survival forest; RF—random forest; SOFA score—the sequential organ failure assessment score; APACHE—the acute physiology and chronic health evaluation; SAPS—the simplified acute physiology score; APS—acute physiology score; LODS—logistic organ dysfunction system; MIMIC—Medical Information Mart for Intensive Care.

## Data Availability

Full dataset can be available upon request. PROSPERO Registration ID: [617678].
